# Women's knowledge about the genitourinary syndrome of menopause: adherence to its treatments in the COVID-19 era in a sample of them: COMEM-GSM study

**DOI:** 10.1186/s12905-021-01548-2

**Published:** 2021-11-30

**Authors:** Laura Baquedano Mainar, Sonia Sánchez Méndez, Peña Dieste Pérez, Mónica Hernández Aragón, Nicolás Mendoza Ladrón de Guevara, the COMEM Study Spanish investigators, L. Baquedano, A. Espiau, S. Ortega, L. Ruiz, M. Lamarca, Y. José, P. Rubio, F. Villalobos, A. Borque, P. Dieste, L. Gabasa, V. Roy, M. J. Puente, M. Chóliz, L. Cotaina, I. Negredo, P. del Tiempo, H. Yagüe, M. Hernández, P. Tajada, M. Fasero, I. Ramirez, L. Gutiérrez, F. Colmenarejo, P. Coronado, T. Aznar, J. Presa, P. Llaneza, R. Sánchez Borrego, S. Palacios, Ana R. Jurado

**Affiliations:** 1 Gynecology Department, Miguel Servet University Hospital, Paseo Isabel La Católica 1-3, 50009 Zaragoza, Spain; 2 Service of Obstetrics and Gynecology, Hospital Universitari General de Catalunya, Barcelona, Spain; 3 Department of Obstetrics and Gynecology, University of Granada, Granada, Spain; 4 Hospital Universitario Miguel Servet of Zaragoza, Zaragoza, Spain; 5 Hospital San Jorge of Huesca, Huesca, Spain; 6 Hospital Universitario La Zarzuela of Madrid, Madrid, Spain; 7 Hospital San Carlos of San Fernando, San Fernando, Spain; 8 Hospital Insular-Materno Infantil of Las Palmas, Las Palmas, Spain; 9 Hospital Quirón Salud of Zaragoza, Zaragoza, Spain; 10 Hospital Clínico San Carlos of Madrid, Madrid, Spain; 11 Hospital Universitario of Castellón, Castellón de la Plana, Spain; 12 Complejo Hospitalario of Jaén, Jaén, Spain; 13 Hospital Universitario Central of Asturias, Oviedo, Spain; 14 Clínica Diatros of Barcelona, Barcelona, Spain; 15 Instituto Palacios of Madrid, Madrid, Spain; 16 European Institute of Sexology, Marbella, Marbella, Spain

**Keywords:** Genitourinary syndrome of menopause, Knowledge, Menopause, COVID-19, Confinement, Adherence

## Abstract

**Objective:**

To study knowledge regarding genitourinary syndrome of menopause (GSM) and the treatments for it and to analyze treatment adherence during the COVID-19 confinement.

**Methods:**

Multi-center observational study including women between 35 and 75 years. An extension study of treatment adherence was conducted during the coronavirus pandemic between March and April 2020.

**Results:**

A sample of 2355 women were included. Vaginal dryness was the most frequently identified symptom (74.3%). Lubricants were the best-known treatments (69.6%), followed by local estrogens (25.7%); 66% of the women did not speak to their gynecologist about sexuality. Comparative analyses were conducted according to age, menopausal status, type of menopause, place of residence, type of health care received and level of education. During the coronavirus confinement period, adherence to treatments for vulvovaginal atrophy was poor in 72.5% asked (n = 204). Reduced sexual activity (*p* > 0.001) and coronavirus diagnosis (*p* = 0.003) were significantly associated with poorer treatment compliance.

**Conclusions:**

There is great lack of knowledge of the treatments used for GSM. Most women do not talk to their gynecologist about sexuality. Adherence to treatments during the coronavirus confinement has been worryingly low.

## Background

Genitourinary syndrome of menopause (GSM) is one of the most frequent complaints referred in transition and postmenopause and it could impact on the quality of life and sexual function of women [1]. Although sexuality is an important element of the lives of many midlife women [2], only a very small percentage of midlife women report having discussed their sexual problems with a healthcare professional [3].

If women are aware of the genital changes produced by hormonal decline and how this can affect their sexuality, it would be easier to identify women with dysfunction or possible symptoms that require treatment. There is an increasing tendency among clinicians to share the treatment options for vulvovaginal atrophy with the patient. The term "empowered therapy" has even been introduced in some publications [4–6]. To make an informed choice, it is important that women have good knowledge about GSM that is free of myths and false beliefs.

This study is part of a population survey known as the COMEM (“COnocimiento de las Mujeres Españolas en Menopausia” in Spanish) study. Data related to menopausal treatments have already been partially published elsewhere [7]. The GSM-related study had two objectives: first, to determine what women know about GSM. Second, considering a small sample of women who previously answered the survey, we aimed to evaluate the adherence to treatment for GSM during the period of confinement that was imposed due to the coronavirus pandemic (COVID-19).

## Methods

This was a multi-center cross-sectional observational study carried out between January and June 2019, using a sample of 2500 women aged between 35 and 75 years. They participants were asked to complete an anonymous and voluntary survey written in simple and clear language.

A team of seven menopause experts selected questions structured in several clinical domains. The study related to therapies in menopause and socio-demographic characteristics of the sample the have been published previously [7]. In the present study, we included the same cohort of women (n = 2355).

The questionnaire was offered to all women in the gynecology consulting-rooms of the participating centers, either they were patients or companions. Women who with physical, psychological or language incapacity to understand the survey questions, or those who did not want to participate were excluded from the study.

In the extension phase of the adherence study, women with treatment for GSM who had done the survey previously, were asked about the adherence and attitude to treatment during the state of alarm for coronavirus infection in Spain (from March 14 to June 21, 2020). Women who discontinued treatment before that time were not included in the extension phase. They were contacted by phone at the control visit after indicating treatment for GSM. Epidemiological data, sexual activity during confinement, type of treatment, dosage, and coronavirus infection data were collected. We evaluated adherence to pharmacological treatment using the Morisky–Green–Levine questionnaire with four items: each item has a dichotomous answer: 'yes' or 'no'. The questions ask if the patient takes the medicine correctly, if she has forgotten and if she abandons it in certain circumstances. The total score ranges from 0 to 4 points. A score of 4 points indicates good adherence to treatment, whilst less than 4 points indicates poor adherence [8].

The research was conducted in accordance with Good Clinical Practice standards and the current revision of the Declaration of Helsinki. The study was approved by the Research Ethics Committee of Aragon Spain (C.I. PI18/374) and of each involved center and was included in the Spanish Menopause Society National Research Network (REIM).

### Statistical analysis

Categorical variables were reported in frequencies and percentages. Quantitative variables were showed using mean and standard deviation. Chi-Square test/Fisher's exact test or Student’s T-test were used for each question of the questionnaire. The Mann–Whitney U test was used for the analysis of dichotomous variables and the Kruskal–Wallis test for non-dichotomous variables when the distribution was non-parametric. The statistically significance was considered as *p* value < 0.05.

Data was collected and codified using IBM Statistics Process Social Sciences 22.0 for Windows (Copyright© SPSS Inc., 2013) for subsequent statistical analysis.

## Results

The answers obtained to the questions of the questionnaire are shown in Table 1. Vaginal dryness was a symptom identified with menopause by 74.3% of the women surveyed and decreased sexual desire by 44.8%. Sexual dysfunction was recognized as a disease associated with menopause by 29%. When asked how menopause affects sexuality, 65% answered that menopause decreases sexual desire. Pain with sexual activity and decreased frequency of sexual intercourse were identify by 34.9% and 27.4% of women asked respectively. Urinary incontinence was associated with menopause by 54% of the women surveyed, while urinary infections in 10.6%.

**Table 1:** GSM knowledge survey and responses given by women surveyed

	n	% of total respondents (n = 2355) (%)
*Which symptoms are associated with menopause?*		
None	14	0.6
Decreased sexual desire	1054	44.8
Vaginal dryness	1749	74.3
Pain during intercourse	822	34.9
Vaginal itchiness	298	12.6
Vulvar itchiness	876	37.2
DK	28	1.2
*Which conditions are associated with menopause?*		
None	99	4.2
Sexual dysfunction	682	29.0
Vaginal atrophy	1766	75.0
Urinary infections	250	10.6
Urinary incontinence	1272	54
DK	88	3.7
*How does menopause affect sexuality?*		
No effects	306	13.0
Decreased sexual desire	1530	65.0
Decreased frequency of sexual activity	646	27.4
DK	186	7.9
*Which of these treatments do you know of for improving vaginal atrophy?*		
Vaginal lubricants	1640	69.6
Vibrators	108	4.6
Laser therapy	73	3.1
Hydrating Creams	558	23.7
Estrogen creams	605	25.7
Sexual intercourse	353	15.0
Hyaluronic acid	172	7.3
DK	443	18.8
*Do you talk to your gynecologist about sexuality?*		
Yes	703	29.9
No	1564	66.4
DK	87	3.7
*If no, indicate the reason(s)*		
Mistrust	49	2.1
Embarrassment	254	10.8
He/she has never asked me	893	57.1
Lack of consultation time	286	18.2
I do not attend this type of clinic	374	24.0
I do not think it is important to him/her	214	9.1

*DK* Don’t know

Among the women surveyed, the most widely known treatments for GSM were lubricants (69.6%) followed by local estrogens (25.7%), whilst 18.8% of the women did not know of any treatment. The benefit of maintaining sexual activity for vaginal atrophy was recognized by 15% of women. A total of 66% of the women surveyed had not discussed the issue of sexuality with their gynecologist, and for most of these women (57.1%) this was because their gynecologist had never asked them (Table 1).

During the coronavirus confinement period, 204 women with treatment for GSM were asked; The median age was 58 years (IQR 54–60) and the age of menopause 50 years (IQR 49–52). The demographic characteristics of the adherence extension phase and the treatments indicated for GSM in the year prior to the declaration of the state of alarm are shown in Table 2. Poor compliance with treatment was observed in 72.5% of the patients included in the study. The most frequent causes of poor adherence were omission (67.7%) and changes in sexual activity (49%). Less sexual activity during the state of alarm and having been diagnosed with coronavirus infection (11.8% of the women in the study) were significantly associated with poorer treatment compliance (*p* < 0.001, *p* = 0.003 respectively) (Table 3).

**Table 2:** Clinical and demographic characteristics of the sample analyzed in the extension study of GSM treatment adherence during COVID-19 confinement

	n (%) (n = 204)
*Age*	
Median (IQR*)	58 (54–60)
*Years of menopause*	
Median (IQR*)	50 (49–52)
*Sexually active*	
Yes	176 (86.3)
No	28 (13.7)
*Level of education*	
Basic-medium	112 (54.9)
University	92 (45.1)
*Sexual activity during confinement*	
Decrease	128 (62.7)
Stable	66 (32.3)
Increase	10 (4.9)
*Indicated treatments*	
Lubricants, moisturizers	48 (23.5)
Local estrogens	120 (58.8)
Prasterone	20 (9.8)
Ospemifene	12 (5.9)
MHT	4 (2)
*Compliance*^a^	
Do you ever forget to take your medication?	52 (25.5)
Do you take the medication at the prescribed times?	96 (47.1)
If you feel well, do you not take it?	52 (25.5)
If it makes you feel worse, do you not take it?	4 (2)
*Good compliance*^b^	
Yes	56 (27.5)
No	148 (72.5)
*Reasons for poor compliance*	
Omission	132 (66.7)
Changes in sexual activity	100 (49)
Fear of going out to purchase it	40 (19.6)
Price	36 (17.6)
Efficacy	20 (9.8)
Other	24 (11.8)
*COVID infection*	
Yes	24 (11.8)
No	180 (88.2)

*MTH* Menopause hormone therapy

^*^IQR: Interquartile range

^a^Questions included in the Morinsky–Green–Levine test [8]

^b^Compliance was considered to be good if the 4 questions were answered as follows: No/Yes/No/No

**Table 3:** Factors related to GSM treatment adherence during COVID-19 confinement

	Good compliance^a^ (n = 56)	Poor compliance^b^ (n = 148)	*p* value
*Age*			
Median (RIQ*)	58 (53.7–60.2)	59 (54–60)	0.988
*Years of menopause*			
Median (RIQ)	50 (49–52)	50 (49–52)	0.693
*Sexually active before confinement*			
Yes	48 (85.7)	128 (86.5)	0.919
No	8 (14.3)	20 (13.5)	
*Level of education*			
Basic-medium	28 (50)	84 (56.8)	0.541
Higher	28 (50)	64 (43.2)	
*Decreased sexual activity*			
Yes	12 (21.4)	100 (67.6)	< 0.001
No	44 (78.6)	48 (32.4)	
*Indicated treatments*			
Lubricants, moisturizers	12 (21.4)	36 (24.3)	0.218
Local estrogens	32 (57.1)	88 (59.5)	
Prasterone	4 (7.1)	16 (10.8)	
Ospemifene	8 (14.3)	4 (2.7)	
MHT	-	4 (2,7)	
*COVID infection*			
Yes	16 (28.6)	8 (5.4)	0.003
No	40 (71.4)	140 (94.6)	

*MTH* menopause hormone therapy

^*^RIQ: Interquartile range

^a^Compliance was considered to be good if the 4 questions of the Morinsky–Green–Levine test were answered correctly, as follows: No/Yes/No/No [8]

^b^Compliance was considered to be poor if any of these 4 questions were answered incorrectly

## Discussion

The main finding of this survey is that the sample of women analyzed have poor knowledge about GSM, particularly about treatment options. In addition, there is also a clear lack of communication between women and their gynecologist in relation to the issue of sexuality. To date, this is the largest sample of women to have been surveyed about their beliefs and knowledge regarding GSM, its treatments, and associated sexuality issues in Spain. In addition, during the extension phase, we have verified that adherence to GSM treatments has been remarkably poor during the coronavirus confinement period. To our knowledge, this is the first study to assess compliance with treatment for GSM during the coronavirus pandemic.

Several studies have analyzed the prevalence and impact of vulvo-vaginal atrophy (VVA) symptoms experienced during menopause [9–12]. The most common symptom among all of these is vaginal dryness, with the European EVES study reporting an estimated prevalence of up to 90%. Dyspareunia is less prevalent (44–72%) but is the most bothersome symptom [9, 10, 12]. Sexuality is the most affected area, as indicated by the VIVA (65%) [11], and the American REVIVE (59%) surveys [9]. In the CLOSER study, the reduction of sexual satisfaction and dyspareunia are causes of significant sexual dysfunction [13]. However, in some of these studies, vaginal atrophy was not recognized as a medical condition by several women, who felt that their concerns had been dismissed as a normal part of aging [9, 10, 14].

When asked about the conditions or symptoms associated with menopause, most of the women in our survey identified vaginal dryness (74.3%); whilst other conditions such as decreased desire or sexual dysfunction were less recognized (44.8% and 29% respectively). The attitudes of the women towards their symptoms are highly variable and may depend on sociocultural and even personality factors [8, 9, 13]. For instance, according to the REVIVE surveys [9, 10], women in Europe appear to be more aware of the fact that vaginal dryness and dyspareunia are menopausal symptoms (53%) than women in the USA (38%).

All these survey-based studies included postmenopausal women, most of them over 45 years of age. The responses of these women about GSM may be conditioned by their own experience and the presence of vulvovaginal symptoms. For this reason, we have thought it interesting to also include younger premenopausal women. This is a new aspect in this type of study, and we think that it better reflects the objective of the study, which is to assess women's knowledge of GSM regardless of their experience.

Another factor that could be associated with the perception of VVA symptoms is communication with the clinicians and the attitude of women in relation to raising the issue of GSM, that is, whether they discuss the symptoms with their health care practitioner during their gynecological appointments. When we specifically asked about sexuality in menopause, only a limited number of women believed that menopause does not affect sexuality (13%). The majority (65%) thought that menopause decreased sexual desire and almost half (34.9%) recognized the symptom of pain during sexual intercourse. According to the AGATA study, 78.7% of 913 patients with VVA had never been questioned by a health care practitioner [15].

In accordance with our findings, the Women’s EMPOWER survey, an internet-based survey of US women with VVA symptoms [4], reported that most women were aware of over-the-counter treatments (vaginal lubricants or moisturizers) but knew rather less about options such as local estrogen treatments. Other surveys evaluated the knowledge, behavior, and attitudes associated with GSM; the authors valued treatments women receive, their satisfaction and concerns, but the treatment options that women are aware of are not discussed in depth [9, 10, 15–17]. This is an important aspect to consider if patients are to choose the most appropriate treatment according to their preferences. It is clear that as gynecologists we must improve the information that we provide to our patients in this regard.

The issue of communication with the clinician in relation to sexuality is particularly interesting. Most of the women in our sample (66.4%) reported that they did not discuss the topic of sexuality with their gynecologist, the reason for which, according to most women (57.1%), was that their clinician had never asked them. In accord with this finding, other studies have shown that women expect their gynecologist to begin the conversation in this regard, and that these women have a strong desire to obtain accurate medical information about VVA [4]. A study was conducted in Spain to evaluate whether actively addressing sexuality in a gynecological consultation with postmenopausal patients improves the diagnosis of sexual problems [18]. A total of 256 postmenopausal women participated in the study. Of these, 12.1% reported a sexual problem during the first 5 min of the interview. The prevalence of patients with a sexual problem increased by 35.9% (from 12.1 to 48.0%) when they were asked about sexuality after 5 min (*p* < 0.0001). These findings suggest that gynecologists should reflect on this issue and consider asking all menopausal women about sexuality.

The clinical efficacy of a treatment over time varies depending on persistence and compliance. Lack of adherence to treatment in chronic diseases is a major problem worldwide [19]. In GSM, both adherence and persistence are low. Among women with GSM who participated in the REVIVE study in Spain, 40% reported having discontinued treatment for GSM [20]. However, our data are even more discouraging. To date, no other study has assessed adherence to GSM treatments during the current coronavirus pandemic. These novel findings should encourage clinicians to reflect on the importance of motivating their patients and emphasizing the benefits of good treatment compliance to avoid unfavorable clinical and economic consequences.

One limitation of our study was the possible selection bias. To minimize this source of bias, the size of the sample was increased and participation in the survey was offered to all women regardless of the reason for their medical visit. Nonetheless, we must assume that the obtained conclusions cannot be extrapolated to the general population and are instead limited to the sample included in the study. Nonetheless, this work might be considered a useful exploratory study that could form the basis for future investigations in the general population. Another possible limitation of our study is related to the choice of questionnaire. There are currently no questionnaires in Spanish that have been sufficiently recognized and validated as an instrument for measuring women's knowledge of menopause. Finally, conducting the Morisky–Green–Levine questionnaire by telephone (which was deemed necessary due to the confinement situation) could also be viewed as a limitation, since the obtained data may be rather different to those that would have been yielded by a self-administered test.

## Conclusions

There is a considerable lack of knowledge regarding the treatments used for GSM. Moreover, most women do not talk to their gynecologist about sexuality issues. Adherence to GSM treatments during the coronavirus confinement has been remarkably low. It is therefore of crucial importance to implement educational and motivational programs and to improve clinician-patient communication about GSM and sexuality during menopause.

## Data Availability

The datasets analysed during the current study available from the corresponding author on request.

## Abbreviations

COMEM: “COnocimiento de las Mujeres Españolas en Menopausia”, in its Spanish acronym
GSM: Genitourinary syndrome of menopause
IQR: Interquartile range
MTH: Menopause hormone therapy
VVA: Vulvo-vaginal atrophy
